# Comparative transcriptome analysis of *Alpinia oxyphylla* Miq. reveals tissue-specific expression of flavonoid biosynthesis genes

**DOI:** 10.1186/s12863-021-00973-4

**Published:** 2021-06-05

**Authors:** Lin Yuan, Kun Pan, Yonghui Li, Bo Yi, Bingmiao Gao

**Affiliations:** 1 Key Laboratory of Tropical Translational Medicine of the Ministry of Education, Hainan Key Laboratory for Research and Development of Tropical Herbs, Hainan Medical University, Haikou, 571199 China; 2 Department of Pharmacy, 928th Hospital of PLA Joint Logistics Support Force, Haikou, 571159 China

**Keywords:** *Alpinia oxyphylla*, Transcriptome analysis, Differentially expressed genes, Secondary metabolites, Flavonoid biosynthesis

## Abstract

**Background:**

*Alpinia oxyphylla* Miq. is an important edible and medicinal herb, and its dried fruits are widely used in traditional herbal medicine. Flavonoids are one of the main chemical compounds in *A. oxyphylla*; however, the genetic and molecular mechanisms of flavonoid biosynthesis are not well understood. We performed transcriptome analysis in the fruit, root, and leaf tissues of *A. oxyphylla* to delineate tissue-specific gene expression and metabolic pathways in this medicinal plant.

**Results:**

In all, 8.85, 10.10, 8.68, 6.89, and 8.51 Gb clean data were obtained for early-, middle-, and late-stage fruits, leaves, and roots, respectively. Furthermore, 50,401 unigenes were grouped into functional categories based on four databases, namely Nr (47,745 unigenes), Uniprot (49,685 unigenes), KOG (20,153 unigenes), and KEGG (27,285 unigenes). A total of 3110 differentially expressed genes (DEGs) and five distinct clusters with similar expression patterns were obtained, in which 27 unigenes encoded 13 key enzymes associated with flavonoid biosynthesis. In particular, 9 DEGs were significantly up-regulated in fruits, whereas expression of 11 DEGs were highly up-regulated in roots, compared with those in leaves.

**Conclusion:**

The DEGs and metabolic pathway related to flavonoids biosynthesis were identified in root, leaf, and different stages of fruits from *A. oxyphylla*. These results provide insights into the molecular mechanism of flavonoid biosynthesis in *A. oxyphylla* and application of genetically engineered varieties of *A. oxyphylla*.

**Supplementary Information:**

The online version contains supplementary material available at 10.1186/s12863-021-00973-4.

## Background

*Alpinia oxyphylla* Miq., a member of the *Zingiberaceae* family, is an important plant species for traditional Chinese medicine, which originates in the Hainan Province and is widely cultivated in southern China [1]. The dried fruits of *A. oxyphylla* are regarded as a valuable drug that has a long clinical history as a well-known constituent of the four southern Chinese medicines in China [2, 3]. The fruits of *A. oxyphylla* are widely used in the treatment of ulcerations, gastralgia, diarrhea, dementia, diabetes, and Alzheimer’s disease [4–9]. Numerous studies have reported that the fruits of *A. oxyphylla* are rich in flavonoids, diarylheptanoids, terpenoids, volatile oils, and steroids and their glycosides [10–13]. Among these compounds, flavonoids and terpenoids are the main active ingredients of *A. oxyphylla* fruits, which have been found to exert various pharmacological activities [13].

Usually, there are variations in the distribution of secondary metabolites in different tissues of higher plants [14–16]. The concentration of chemical constituents was comparable in roots and leaves of *A. oxyphylla*, but was significantly higher in fruits [17]. In addition, the content of chemical compounds in the fruits of *A. oxyphylla* harvested at different times indicates that the 45-day harvested fruit had the highest content of chemicals [17, 18] The metabolic processes and regulatory mechanisms of these chemical compounds in different tissues and fruits at different stages have not yet been elucidated.

The transcriptome is a complete set of RNA transcripts in a cell at a specific developmental stage, and provides information on gene expression and regulation related to a variety of cellular processes including secondary metabolite biosynthesis [19, 20]. With the development of next-generation sequencing, RNA sequencing is an effective method for investigating the metabolic pathways influenced by active ingredients and associated gene expression in different tissues or samples, such as flavonoid biosynthesis in *Ampelopsis megalophylla* [21], terpenoids metabolism in ginseng roots [22] and polysaccharide and alkaloid content in *Dendrobium* [23]. To date, there are no studies on the genetic modification of *A. oxyphylla* either toward increased production of secondary metabolites or biomass accumulation. Therefore, it is important to explore the whole genome transcriptome of *A. oxyphylla* to identify candidate genes contributing to metabolic processes and regulatory mechanisms.

In this study, the differentially expressed genes (DEGs) and metabolic pathway related to flavonoids biosynthesis were identified in root, leaf, and different stages of fruits from *A. oxyphylla*. Therefore, the results of this study may serve as a significant resource for developing genetically engineered varieties of *A. oxyphylla* with improved quality and yield.

## Results

### De novo assembly

The three tissue samples (fruits of different developmental stages, leaves, and roots) of *A. oxyphylla* were sequenced using Illumina HiSeq 4000 which generated approximately 29.50, 33.67, 28.93, 22.98, and 27.84 million pair-end short reads with a length of 150 bp for early-fruits, middle-fruits, late-fruits, leaves, and roots, respectively. After filtering out low-quality reads and adapters, we obtained 8.85, 10.10, 8.68, 6.89, and 8.51 Gb clean data for each sample, and the clean data ratio were estimated to be 99.84, 99.85, 99.84, 99.80, and 99.86%, respectively (Table 1). The lllumina reads have been deposited in the Sequence Read Archive (SRA) database at NCBI (https://www.ncbi.nlm.nih.gov/sra) and thier accession numbers were SRX6686137, SRX6686136, SRX6686135, SRX6686134, and SRX6686133, respectively. De novo assembly of the short reads generated 262,114 contigs and 140,126 unigenes for the whole transcriptome, and N50 was calculated to be 1567 bp and 1073 bp and the mean lengths were 916 bp and 658 bp. The average GC content of contigs and unigenes for the *A. oxyphylla* transcriptome were 43.76 and 43.78%, respectively (Table 1).

**Table 1:** Sequencing statistics and assembly summary for the fruits, leaves, and roots of *A. oxyphylla*

Samples	Fruits			Leaves	Roots
	Early	Middle	Late		
	**Raw data**				
Total Reads	29,496,176	33,671,483	28,927,107	22,975,241	27,836,177
Total length (bp)	8,848,852,800	10,101,444,900	8,678,132,100	6,892,572,300	8,350,853,100
Read length (bp)	150	150	150	150	150
	**Clean data**				
Total Reads	29,448,034	33,622,040	28,882,070	22,928,184	27,796,543
Total length (bp)	8,834,410,200	10,086,612,000	8,664,621,000	6,878,455,200	8,338,962,900
Clean data ratio	99.84%	99.85%	99.84%	99.80%	99.86%
	**Contigs**				
Total Number	262,114				
Total Length (bp)	240,350,061				
Mean Length (bp)	916				
N50 (bp)	1567				
N70 (bp)	939				
N90 (bp)	352				
GC Content	43.76%				
	**Unigenes**				
Total Number	140,126				
Total Length (bp)	92,262,411				
Mean Length (bp)	658				
N50 (bp)	1073				
N70 (bp)	507				
N90 (bp)	263				
GC Content	43.78%				

### Functional annotation and classification

**Fig. 1:**
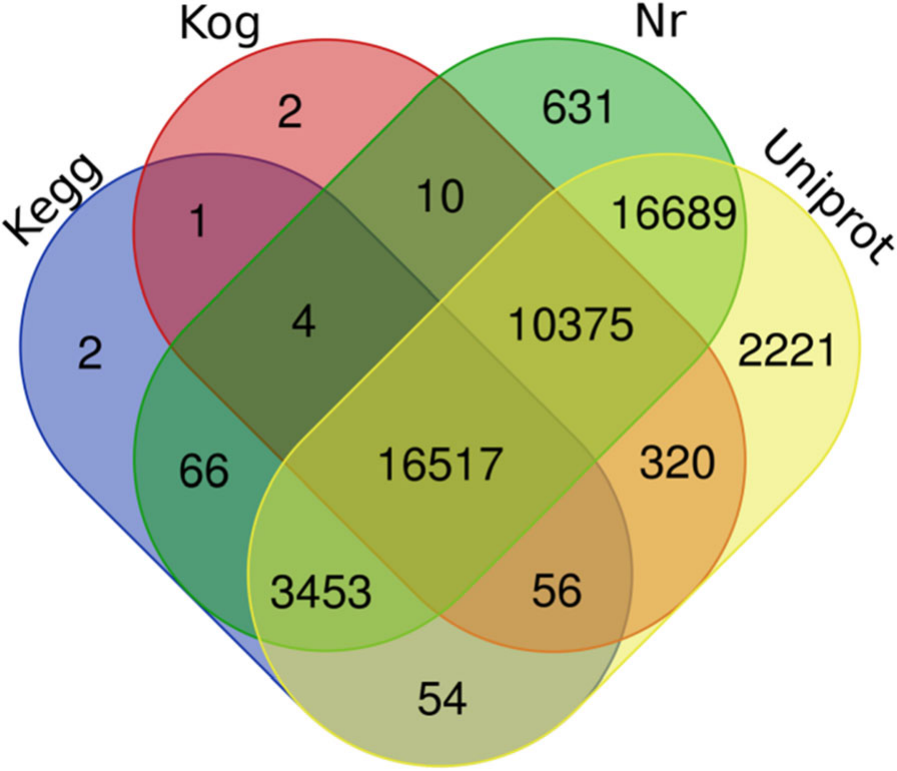
Venn diagram describing the unigenes annotated by four different databases. The integration of unique similarity search results against the NCBI non-redundant protein (Nr), Universal Protein (Uniport), EuKaryotic Orthologous Groups (KOG), and Kyoto Encyclopedia of Genes and Genomes (KEGG) databases

To investigate the function of unigenes, annotation was performed based on four databases. A total of 50,401 unigenes were grouped into the databases, non-redundant protein (Nr) (47,745 unigenes), Universal Protein (Uniport) (49,685 unigenes), EuKaryotic Orthologous Groups (KOG) (20,153 unigenes), and Kyoto Encyclopedia of Genes and Genomes (KEGG) (27,285 unigenes), respectively, while an additional 89,725 unigenes were not found in these databases. A detailed comparison of the unigenes annotated by four different databases are illustrated in Fig. 1.

GO analysis illustrated that 37,555 unigenes of *A. oxyphylla* were annotated into three categories: molecular function (30,356), cellular component (20,203), and biological process (26,368), respectively (Supplementary Fig. 1 in Additional file 1). The binding (19,730) and catalytic activity (17,452) functional groups were the most prominent molecular functions. A total of 20,153 unigenes of *A. oxyphylla* were further annotated and grouped into 25 molecular families in KOG database (Supplementary Fig. 2 in Additional file 1). These molecular families were grouped into four categories: information storage and processing (5575), cellular processes and signaling (7377), metabolism (6180), and poorly characterized (5803). For KEGG analysis, 29,211 unigenes of *A. oxyphylla* had significant matches in the database and were assigned to five primary categories: cellular processes (3324), environmental information processing (2571), genetic information processing (5073), metabolism (13,599), and organismal systems (4644) (Supplementary Fig. 3 in Additional file 1). A majority of unigenes were assigned to metabolism, and global and overview maps had the highest number of annotated unigenes (5005).

### Differential gene expression analysis

**Fig. 2:**
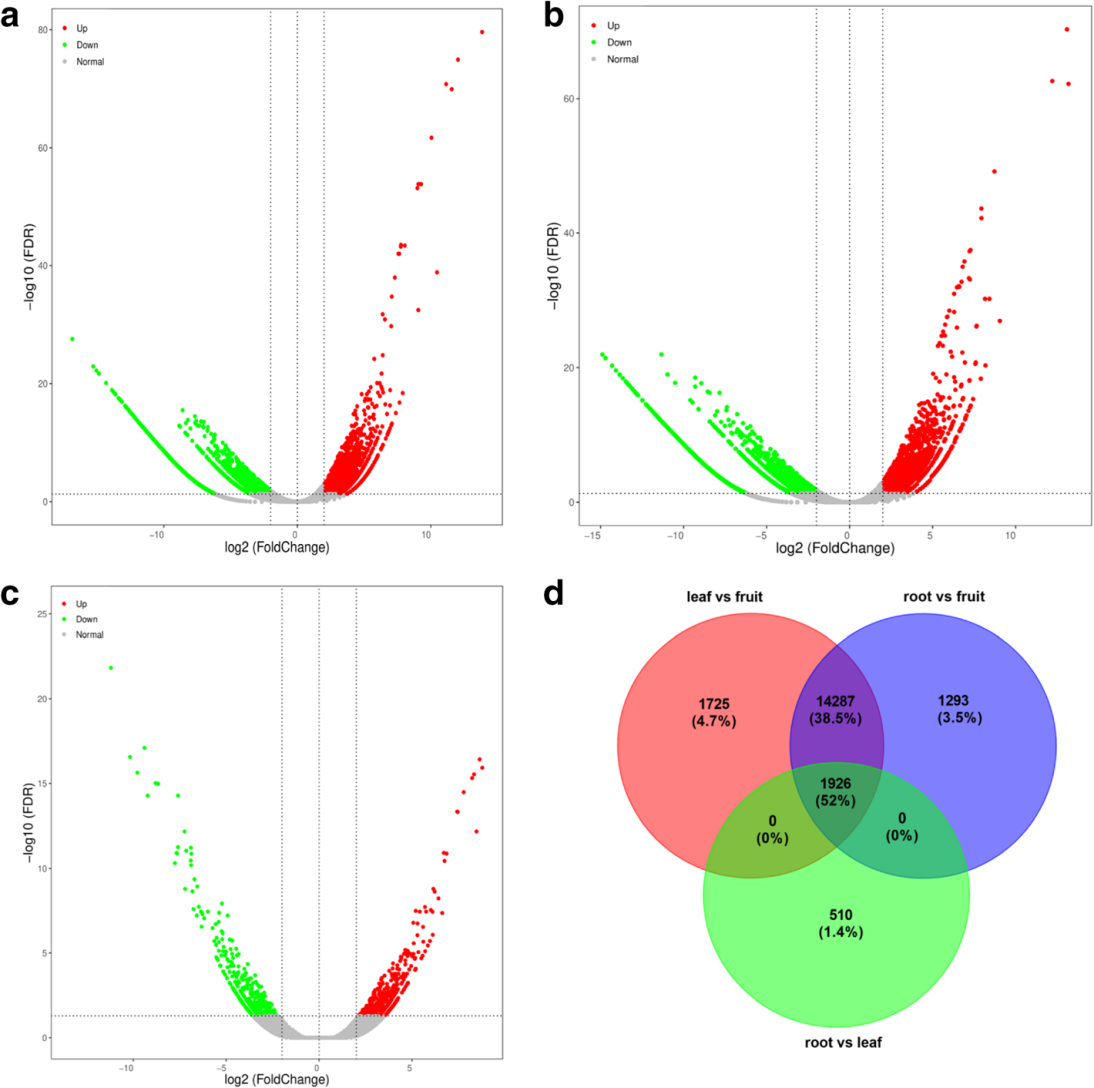
Volcano plots of the differentially expressed genes (DEGs) in the comparison group of (**a**) leaf vs. fruit, (**b**) root vs fruit, and (**c**) root vs. leaf. (**d**) Venn diagram of DEGs in three different comparisons groups represented by three circles. The overlapping parts of the circles represent the number of DEGs in common in the comparison groups

There were 35,278 DEGs identified between the leaf vs fruit sample, including 15,063 up-regulated and 20,215 down-regulated DEGs in fruit (Fig. 2a). A total of 34,846 DEGs were identified between root vs. fruit sample, including 14,807 up-regulated and 20,039 down-regulated DEGs in fruit (Fig. 2b). There were 19,776 DEGs between root vs. leaf sample, out of which 8797 were up-regulated and 10,979 were down-regulated in leaf (Fig. 2c). Using a Venn diagram, we compared the data sets from the three comparison groups (leaf vs. fruit, root vs. fruit, and root vs. leaf). In this comparison, 19,266 DEGs were identified as common (Fig. 2d) to all three groups. A total of 16,213 DEGs were identified in both “leaf vs. fruit” and “root vs. fruit” comparisons; 19,266 DEGs were identified in both “leaf vs. fruit” and “root vs. leaf” comparisons; while 19,266 DEGs were identified in both “root vs. fruit” and “root vs. leaf” comparisons.

### Cluster and KEGG enrichment analysis of DEGs

**Fig. 3:**
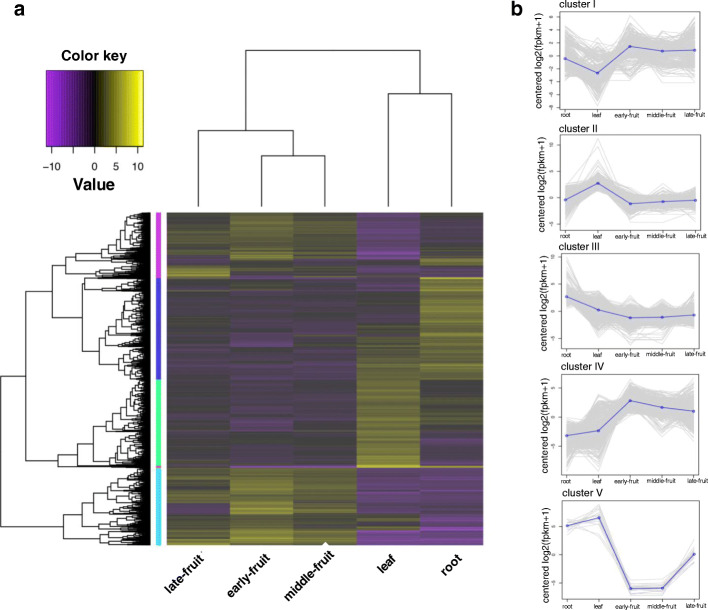
Cluster analysis of DEGs (**a**) Heat-map showing the expression of DEGs using RNA-seq data derived from mean value of three replicates of each sample based on log 2 (FPKM) values. Color code indicates expression levels. Similarity between samples and unigenes with hierarchical clustering is shown above and on the left of the heatmap, respectively. (**b**) Cluster analysis of all DEGs. The y-axis in each graph represents the mean-centered log2 (FPKM+ 1) value. Expression of a single gene is plotted in gray, while the mean expression of the genes in each cluster is plotted in blue

**Fig. 4:**
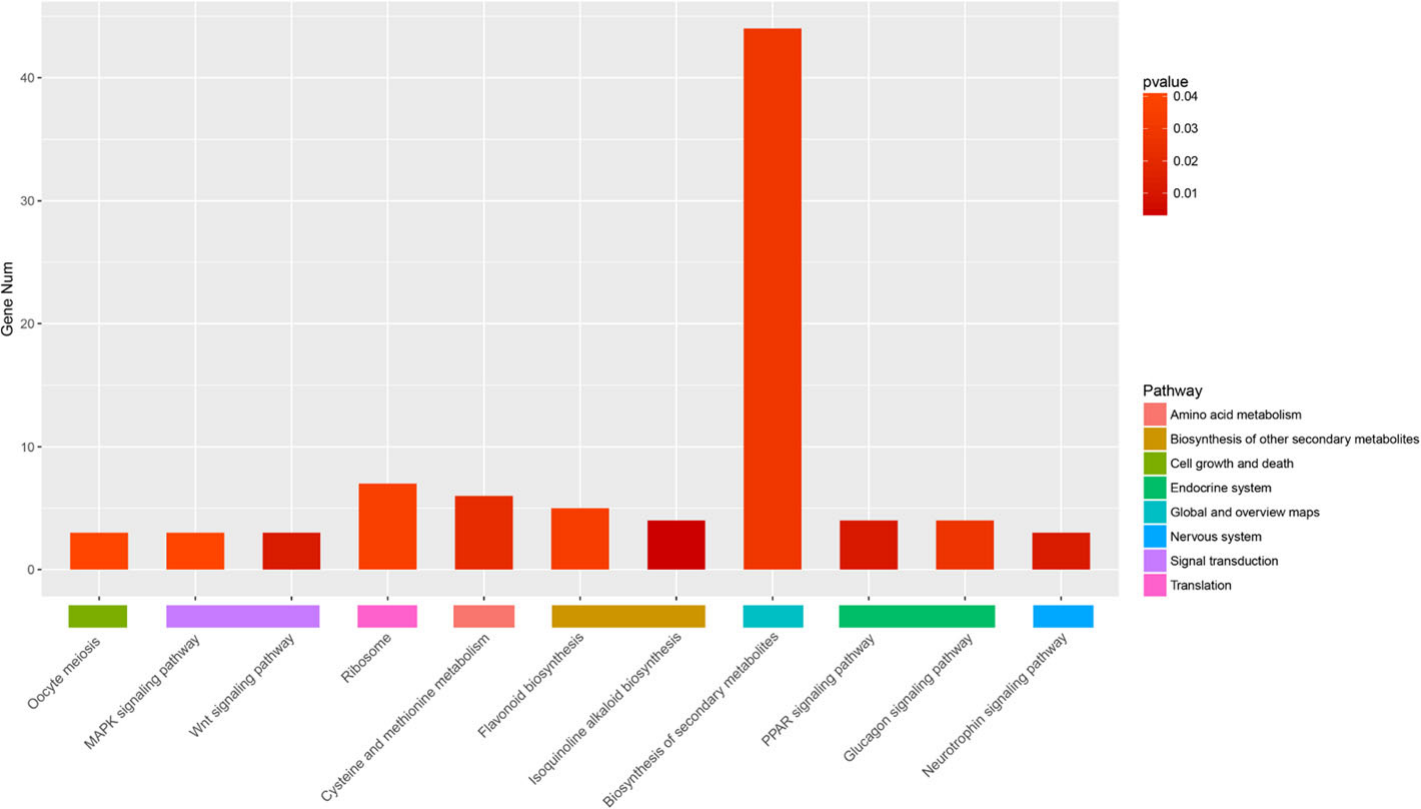
Distribution map of DEGs in cluster I signaling pathway

To investigate the expression trends of DEGs in different tissues, we performed a cluster analysis using normalized expression values from each individual replicate of five different samples of *A. oxyphylla*. As a result, a total of 3110 DEGs and five distinct clusters with similar expression patterns were obtained, containing 606, 807, 954, 725, and 18 genes, respectively (Fig. 3a). As shown in Fig. 3b, the expression level of cluster I (606) and cluster IV (725) genes in fruits of *A. oxyphylla* were higher than in roots and leaves, and the expression levels of cluster II (807), cluster III (954), and cluster V (18) in fruits were lower than in roots and leaves. The secondary metabolites in fruits are higher than roots and leaves, for instance, flavonoids in fruits are 1000 times higher than roots and leaves [17]. Therefore, the DEGs related to secondary metabolite biosynthesis should be in cluster I and cluster IV. Signal pathway analysis of DEGs in the five clusters showed that cluster I contains DEGs involved in flavonoid biosynthesis, isoquinoline alkaloid biosynthesis, and biosynthesis of secondary metabolites (Fig. 4).

Through further comparative analysis, there were 35 and 44 DEGs related to secondary metabolites in root vs fruit and leaf vs fruit, repetively (Table 2). These DEGs were mainly distributed in phenylpropanoid, flavonoid and isoquinoline alkaloid biosynthesis pathways. For phenylpropanoid biosynthesis pathways, 14 DEGs were up-regulated and 3 DEGs were down-regulated in root vs fruit, and 19 DEGs were up-regulated, 5 DEGs were down regulated in leaf vs fruit. It is noteworthy that all the 8 DEGs mapped to flavonoids biosynthesis, and they were both up-regulated in leaf vs fruit (Table 2). In addition, 2 DEGs were up-regulated in anthocyanin biosynthesis, 3 DEGs were down-regulated in diarylheptanoid and gingerol biosynthesis, 1 DEGs were up-regulated and 2 DEGs were down-regulated in sesquiterpenoid and triterpenoid biosynthesis. In conclusion, phenylpropanoid, flavonoids and isoquinoline alkaloid biosynthesis related DEGs were significantly up-regulated, while diarylheptanoid, gingerol, sesquiterpenoid, triterpenoid and carotenoid biosynthesis related DEGs were down-regulated in fruits compared with roots and leaves.

**Table 2:** Comparative analysis of gene expression regulation of secondary metabolites biosynthesis in fruits, roots and leaves

Group	ROOT	Second	mapID	Description	DEGs	up-gene in Fruit	down-gene in fruit
root vs fruit	metabolism	biosynthesis of other secondary metabolites	map00940	phenylpropanoid biosynthesis	35	14	3
			map00942	anthocyanin biosynthesis		2	0
			map00945	stilbenoid, diarylheptanoid and gingerol biosynthesis		0	3
		metabolism of terpenoids and polyketides	map00909	sesquiterpenoid and triterpenoid biosynthesis		1	2
leaf vs fruit	metabolism	biosynthesis of other secondary metabolites	map00940	phenylpropanoid biosynthesis	44	19	5
			map00941	flavonoid biosynthesis		8	0
			map00950	isoquinoline alkaloid biosynthesis		7	0
		metabolism of terpenoids and polyketides	map00906	carotenoid biosynthesis		0	5

### Candidate genes associated with flavonoid biosynthesis

**Fig. 5:**
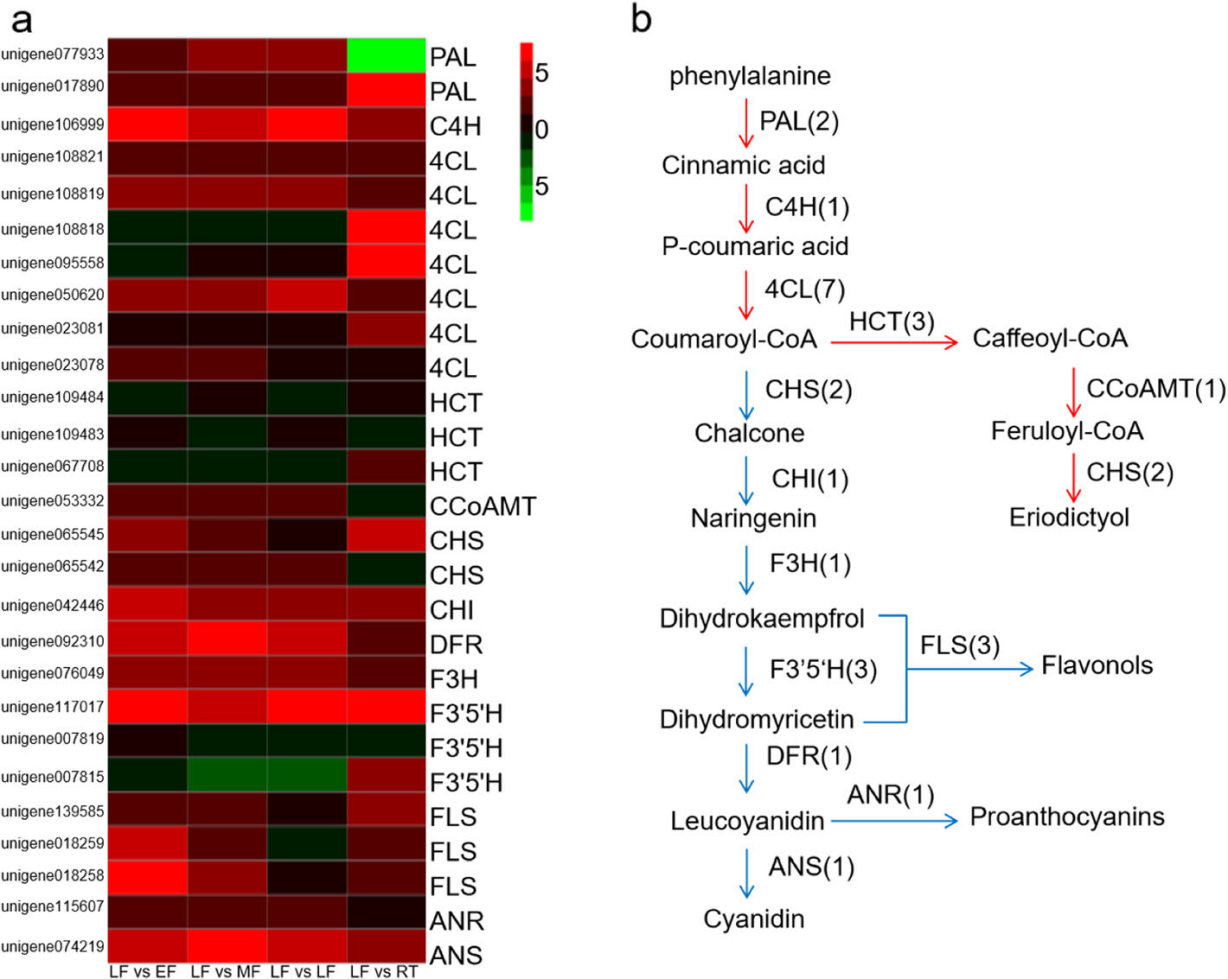
Putative flavonoid biosynthesis pathway in *A. oxyphylla*. (**a**) Expression level of candidate *A. oxyphylla* unigenes coding for key enzymes involved in flavonoid biosynthesis pathways. Green and red colors are used to represent low-to-high expression levels (mean centered log_2_-transformed FPKM values). (**b**) Pathway for flavonoid biosynthesis. The numbers in brackets following each gene name indicate the number of *A. oxyphylla* unigenes corresponding to that gene. Enzyme abbreviations are as follows: PAL, phenylalanine ammonia-lyase; C4H, cinnamate 4-hydroxylase; CHS, chalcone synthase; CCoAMT, Caffeoyl Co-A transferase; 4CL, 4-coumarate-CoA ligase; CHI, chalcone isomerase; F3H, flavanone 3-hydroxylase; F3’5’H, flavonoid-3′, 5′-hydroxylase; DFR, dihydroflavonol-4-reductase; ANR, anthocyanidin reductase; ANS, anthocyanidin synthase

Flavonoids are one of the main chemical compounds found in *A. oxyphylla* and are important for evaluating its quality [18]. To understand the regulation of flavonoid biosynthesis in *A. oxyphylla*, key regulatory genes involved in the pathways for phenylpropanoid and flavonoid biosynthesis were identified in this study. Twenty-seven unigenes encoding 13 key enzymes observed in this study were mostly associated with biosynthesis of flavonoids. Furthermore, results of the microarray analysis of tissue-specific transcriptomes demonstrated that the majority of genes encoding enzymes in the biosynthesis of flavonoids were expressed preferentially in the fruit of *A. oxyphylla* (Fig. 5a). In particular, 9 DEGs, including chalcone synthase (CHS), chalcone isomerase (CHI), flavanone 3-hydroxylase (F3H), flavonol synthase (FLS), anthocyanidin synthase (ANS), dihydroflavonol-4-reductase (DFR), and anthocyanidin reductase (ANR) unigenes, were significantly up-regulated in fruits, whereas expression of 11 DEGs including flavonoid-3′, 5′-hydroxylase (F3’5’H), hydroxycinnamoyl transferase (HCT), Caffeoyl Co-A transferase (CCoAMT), 4-coumarate-CoA ligase (4CL) and phenylalanine ammonia-lyase (PAL), were highly up-regulated in roots. However, the flavonoid biosynthesis associated genes exhibited low expression levels in leaves, particularly 4CL and FLS displayed an expression value of 0 (Supplementary Table 1 in Additional file 2). In previous studies, flavonoids are found in high concentrations in fruits, followed by roots, and are found in the lowest concentrations in leaves [17]. Expression analysis of flavonoid biosynthesis genes in the present study also showed a similar trend. The putative flavonoid synthesis pathway is shown in Fig. 5b. Flavonoids are synthesized via the phenylpropanoid pathway and are converted from phenylalanine to chalcone by the enzymes phenylalanine ammonia-lyase (PAL), cinnamate 4-hydroxylase (C4H), 4CL, and CHS. CHI catalyzes the isomerization of chalcones into flavanone. Flavanone can be converted either to flavonols through the subsequent action of F3H and FLS, or to flavone through the action of DFR and LAR. However, no unigene coding for flavone synthase (FNS) was detected in the transcriptome analysis. A similar situation has been reported in the transcriptome sequencing of other plants such as *Sophora japonica*, which may be attributed to the fact that FNS genes are short fragments without sequence similarity [24].

## Discussion

There are about 250 species of *Alpinia* plants distributed in tropical Asia [25]. The roots and fruits of *Alpinia* plants are often used for medicinal applications [2, 26]. The capsular fruit of *A. oxyphylla* has been used as a medicinal constituent or health supplement for centuries as one of the four famous southern Chinese medicines [2, 3]. Studies in natural product chemistry reveal that the capsular fruit, root, and leaf contain flavonoids, sesquiterpenes, diarylheptanoids, essential oils, glycosides, and steroids [14, 17]. The main chemical components of *A. oxyphylla* flavonoids comprise of tectochrysin, izalpinin, chrysin, and kaempferide, of which tectochrysin is the second most abundant flavonoid concentrated in fruits [11]. Therefore, flavonoids are one of the most important active chemical components in *A. oxyphylla* and are important for evaluating its quality. However, the molecular mechanism of tissue-specific flavonoid biosynthesis and accumulation in *A. oxyphylla* remains largely unexplored.

In this study, we collected three tissue samples (fruits of different developmental stages, leaves, and roots) of *A. oxyphylla* and performed a comparative transcriptome analysis, with a particular focus on flavonoid biosynthesis genes. To analyze if the gene expression of biosynthetic genes also follow this pattern, high-throughput transcriptome sequencing technology was employed. Indeed, transcriptional analysis showed that a large number of transcripts exhibited a tissue-specific expression. The number of DEGs in the ‘leaf vs. fruit’ and ‘root vs. fruit’ comparison groups was higher than that in the ‘root vs. leaf’ comparison group. These results suggest that the medicinal properties and associated biological processes are concentrated in the fruits of *A. oxyphylla*. To investigate the trends of DEGs in gene expression, we performed a cluster analysis using normalized expression values from each individual replicate of five different samples of *A. oxyphylla*. A total of 3110 DEGs were divided into five distinct clusters according to their expression patterns. Further analysis showed that only the cluster I of DEGs were related to flavonoid biosynthesis, isoquinoline alkaloid biosynthesis and biosynthesis of secondary metabolites, and the expression level in fruits was significantly higher than that in leaves and roots. The enriched KEGG pathways results showed that all the DEGs related to flavonoid biosynthesis were up-regulated, and most of the DEGs involved in phenylpropanoid biosynthesis were also up-regulated, but the DEGs related to stilbenoid, diarylheptanoid and gingerol biosynthesis were down-regulated in fruits, indicating that flavonoids were the main secondary metabolites. The characterized flavonoids, including tectochrysin, izalpinin, chrysin, and kaempferide, are found in greatest concentrations in fruits, followed by roots, and are found in the lowest concentrations in leaves [17]. Therefore, the expression level of flavonoid related genes was consistent with that of chemical components in different tissues of *A. oxyphylla*.

The biosynthesis of flavonoids has been reported in many other medicinal plants such as *Astragalus membranaceus* var. mongholicus, *Apocynum venetum,* and *Eucommia ulmoides*, and phenylpropanoid biosynthesis is the common core pathway for the synthesis of flavonoids [27–29]. The first step in flavonoid biosynthesis is regulated by enzymes (PAL, C4H, and 4CL) in the phenylpropanoid pathway. The substrate 4-coumaroyl-CoA is converted into chalcone by CHS in the first rate-limiting step of flavonoid biosynthesis [30]. Next, different flavonoid subgroups are synthesized through modification of the molecular backbone, which is controlled by flavonoid, flavone and flavonol biosynthesis enzymes such as HCT, CCoAMT, CHS, CHI, F3H, F3′,5′H, DFR, ANR, and ANS [29–32]. In this study, homologous unigenes and the expression levels of these genes were investigated in samples of different tissues from *A. oxyphylla*.

Interestingly, DEGs encoding CHS, CHI, F3H, FLS, ANS, DFR and ANR were highly expressed in the samples from fruits than the other two tissues, and DEGs encoding PAL, 4CL, HCT, CCoAMT, and F3’5’H were highly expressed in the samples from roots than the other two tissues. It is noteworthy that PAL and 4CL display high expression in roots, but the flavonoids are not concentrated in the root [17]. It is speculated that in the initial stages of flavonoid synthesis, phenylpropanoid biosynthesis pathway initiates synthesis of substrates in the root, part of which is converted into eriodictyol by HCT, CCoAMT, and F3’5’H, and the rest is transported to the fruit, where it is modified and processed by CHS, CHI, F3H, FLS, ANS, DFR, and ANR to form flavonoids, flavones, and flavonols (Fig. 5). Therefore, it reasonable to primarily utilize fruits of *A. oxyphylla* as components of traditional medicine, rather than the root as done in species such as *A. officinarum*. These results provide insights into the molecular processes of flavonoid biosynthesis in *A. oxyphylla* and offer a significant resource for the application of genetic engineering to develop varieties of *A. oxyphylla* with improved quality.

## Conclusions

In this study, a total of 3110 DEGs and five distinct clusters with similar expression patterns were obtained, in which 27 unigenes encoded 13 key enzymes associated with flavonoid biosynthesis. In particular, 9 DEGs were significantly up-regulated in fruits, whereas expression of 11 DEGs were highly up-regulated in roots, compared with those in leaves. In summary, The DEGs and metabolic pathway related to flavonoids biosynthesis were identified in root, leaf, and different stages of fruits from *A. oxyphylla*. These results provide insights into the molecular mechanism of flavonoid biosynthesis in *A. oxyphylla* and application of genetically engineered varieties of *A. oxyphylla*.

## Methods

### Plant material

*A. oxyphylla* were collected from cultivated fields in Baisha County, Hainan Province, China (N.109.437569, E.19.19680). The sample was identified by Kun Pan and deposited at the Key Laboratory of Tropical Translational Medicine of the Ministry of Education, Hainan Medical University, Haikou, Hainan, China. The specimen accession number was CHMU0123. The fruits were sampled at the following three developmental stages: early-fruit (15 days), middle-fruit (30 days) and late-fruit (45 days). Fresh *A. oxyphylla* fruits were obtained from the three plants simultaneously during each phase. Then, the materials of same phase were mixed for further experiments. After harvesting the fruit, the leaves and roots were obtained from the same plant. All the samples of *A. oxyphylla* were immediately frozen in liquid nitrogen and stored at − 80 °C prior to processing.

### RNA sequencing and De novo assembly

The total RNA was extracted from different plant tissues using the RNAprep Pure Plant Kit (Tiangen, Beijing, China) as per the standard protocol [33]. The RNA concentration and quantity were assessed using the Nanodrop 2000 spectrometer (Thermo Fisher Scientific, Wilmington, DE, USA) and Agilent Bioanalyzer 2100 system (Agilent Technologies, Santa Clara, CA, USA). A Stranded Total RNA Library Prep Kit (Illumina, Inc., San Diego, AR, USA) was used for cDNA library construction and normalization. The cDNA library was sequenced using Illumina HiSeq 4000 as per standard protocol. Raw reads were filtered by removing the adapter and low-quality sequences to produce high-quality clean reads and the reads were assembled to generate unigene libraries. Trinity software (v.2.8.5, the Broad Institute, Cambridge, MA, USA) was used to assemble the clean data into unigenes according to a basic group quality score of more than Q30 [34].

### Functional annotation

Function annotation of the assembled unigenes were obtained from public databases NCBI Nr (http://www.ncbi.nlm.nih.gov), Uniport (https://www.uniprot.org/), KOG (ftp://ftp.ncbi.nih.gov/pub/ COG/KOG), and KEGG classifications (http://www.genome.jp/kegg/).

### Analysis of DEGs

Unigene expression level was calculated using the fragments per kilobase of transcript per million mapped (FPKM) method. The DEGs were screened using the edgeR package with the threshold set as described previously [35]. GO and KEGG enrichment analysis of the identified DEGs was performed using the GOAtools version 0.5.9 (https://github.com/tanghaibao/Goatools) and KOBAS version 2.0.12 with default settings, respectively. The corrected *p*-value for identifying significant differences in expression was calculated and adjusted by the hypergeometric Fisher exact test. GO terms with a corrected p-value≤0.05 were considered to be significantly enriched. Next, we employed the same method for KEGG pathway functional enrichment analysis of DEGs.

## Supplementary Information

**Additional file 1: Supplementary Fig. 1.** GO classification of assembled unigenes of *A. oxyphylla*. **Supplementary Fig. 2.** KOG classification of assembled unigenes of *A. oxyphylla.* **Supplementary Fig. 3.** KEGG functional classification of assembled unigenes of *A. oxyphylla*.

**Additional file 2: Supplementary Table 1.** Expression level of candidate *A. oxyphylla* unigenes coding for key enzymes involved in flavonoid biosynthesis pathways.

## Data Availability

The lllumina reads have been deposited in the Sequence Read Archive (SRA) database at NCBI (https://www.ncbi.nlm.nih.gov/sra) and are available under study accession numbers: SRX6686137, SRX6686136, SRX6686135, SRX6686134, and SRX6686133.

## Abbreviations

DEGs: Differentially expressed genes
Nr: Non-redundant protein
Uniport: Universal Protein
KOG: EuKaryotic Orthologous Groups
KEGG: Kyoto Encyclopedia of Genes and Genomes
FPKM: Fragments per kilobase of transcript per million mapped
HCT: Hydroxycinnamoyl transferase
FNS: Flavone synthase
PAL: Phenylalanine ammonia-lyase
C4H: Cinnamate 4-hydroxylase
CHS: Chalcone synthase
CCoAMT: Caffeoyl Co-A transferase
4CL: 4-coumarate-CoA ligase
CHI: Chalcone isomerase
F3H: Flavanone 3-hydroxylase
F3’5’H: Flavonoid-3′,5′-hydroxylase
DFR: Dihydroflavonol-4-reductase
ANR: Anthocyanidin reductase
ANS: Anthocyanidin synthase
