# Supplementary material for: Comparative transcriptome analysis of Alpinia oxyphylla Miq. reveals tissue-specific expression of flavonoid biosynthesis genes
Source: BMC Genom Data. 2021 Jun 5;22:19. doi: 10.1186/s12863-021-00973-4 (PMC8180045; doi:10.1186/s12863-021-00973-4)
Supplement: Supplementary file 1 — Additional file 1: Supplementary Fig. 1. GO classification of assembled unigenes of A. oxyphylla. Supplementary Fig. 2. KOG classification of assembled unigenes of A. oxyphylla. Supplementary Fig. 3. KEGG functional classification of assembled unigenes of A. oxyphylla. [file 12863_2021_973_MOESM1_ESM.docx]

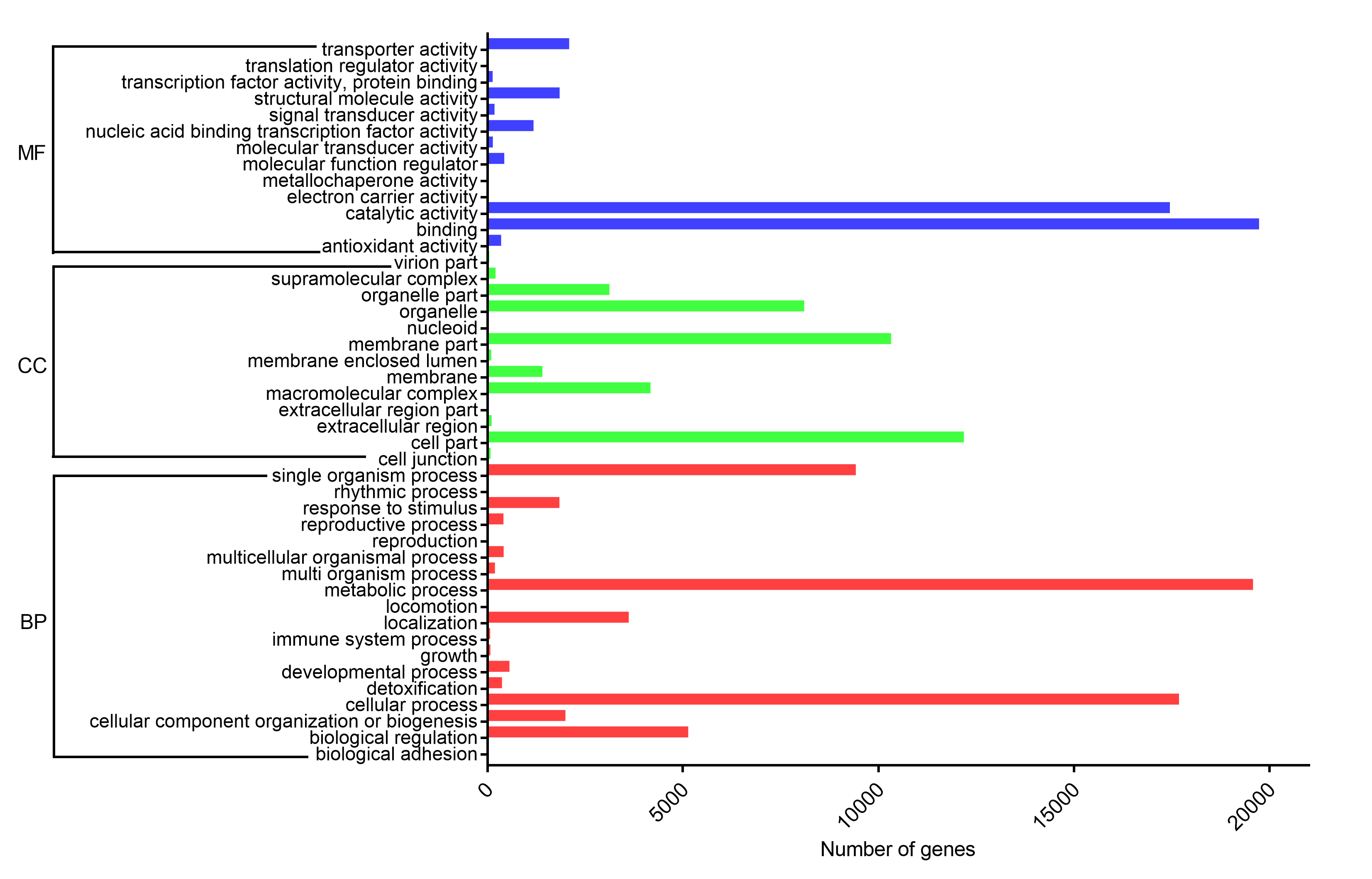


**Additional file 1 Supplementary Fig. 1.** GO classification of assembled unigenes of *A. Oxyphylla*. A total of 218,989 unigenes were annotated into three categories: molecular function (MF, blue), cellular component (CC, green) and biological process (BP, red).


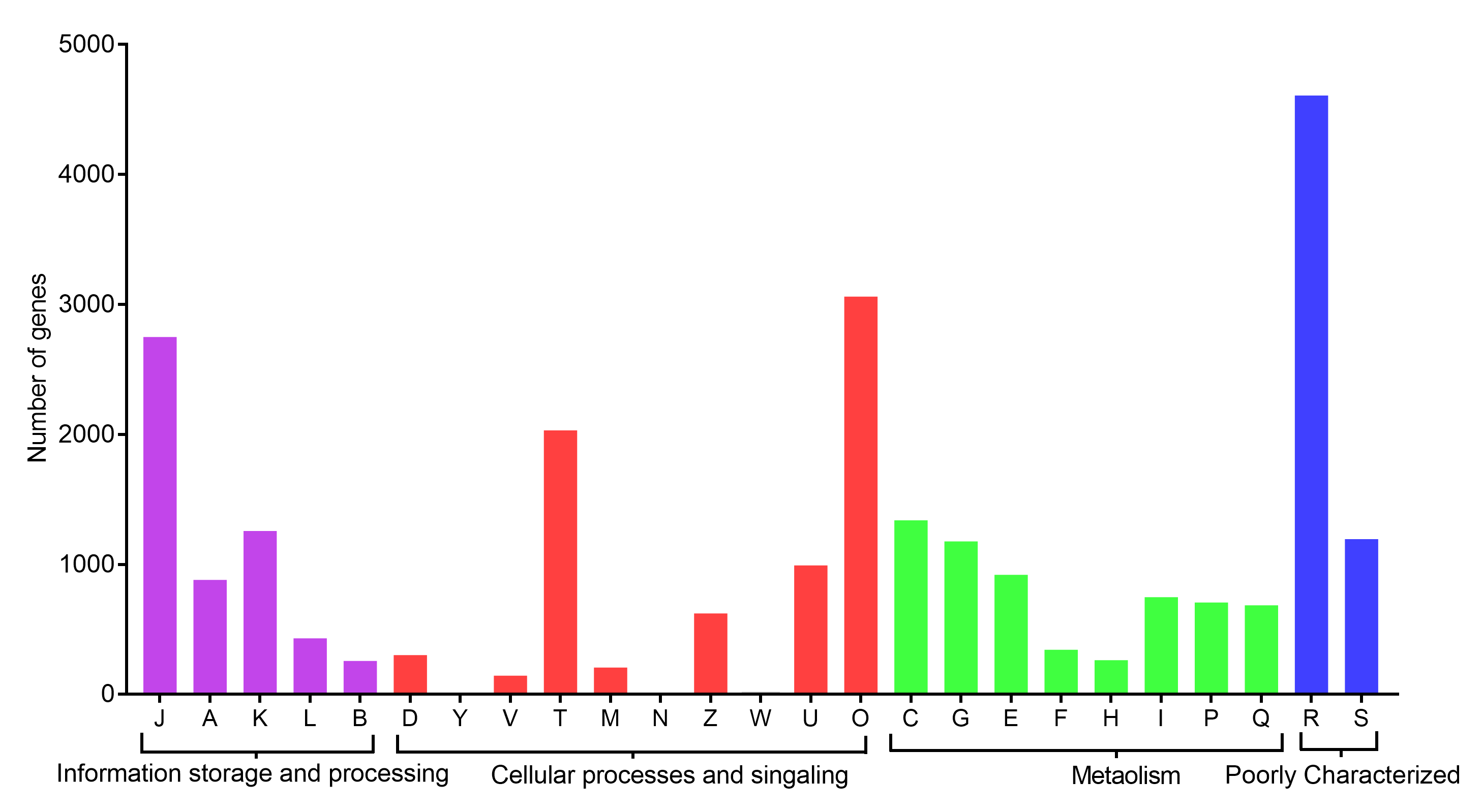


**Additional file 1 Supplementary Fig. 2.** KOG classification of assembled unigenes of A. Oxyphylla. [J] Translation, ribosomal structure and biogenesis; [A] RNA processing and modification; [K] Transcription; [L] Replication; recombination and repair; [B] Chromatin structure and dynamics; [D] Cell cycle control, cell division, chromosome partitioning; [Y] Nuclear structure; [V] Defense mechanisms; [T] Signal transduction mechanisms; [M] Cell wall/membrane/envelope biogenesis; [N] Cell motility; [Z] Cytoskeleton; [W] Extracellular structures; [U] Intracellular trafficking, secretion and vesicular transport; [O] Posttranslational modification, protein turnover, chaperones; [C] Energy production and conversion; [G] Carbohydrate transport and metabolism; [E] Amino acid transport and metabolism; [F] Nucleotide transport and metabolism; [H] Coenzyme transport and metabolism; [I] Lipid transport and metabolism; [P] Inorganic ion transport and metabolism; [Q] Secondary metabolites biosynthesis, transport and catabolism; [R] General function prediction only; [S] Function unknown.
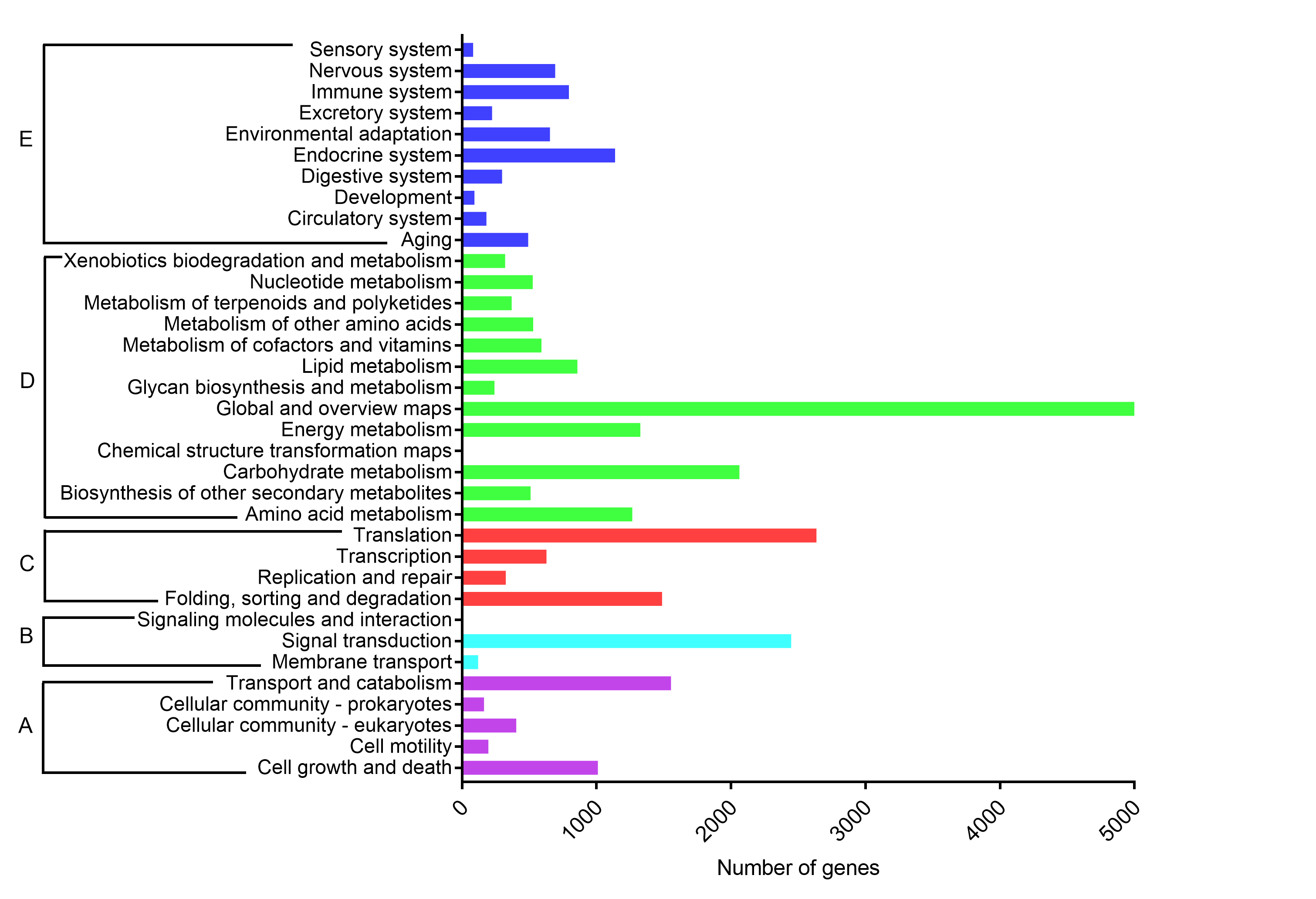


**Additional file 1 Supplementary Fig. 3.** KEGG functional classification of assembled unigenes of *A. Oxyphylla*. The unigenes were divided into five primary categories: (A) cellular processes, (B) environmental information processing, (C) genetic information processing, (D) metabolism, and (E) organismal systems. The x-axis represents the number of unigenes, whereas the y-axis represents the functional categories.
