# Supplementary material for: Comparative transcriptome analysis of Alpinia oxyphylla Miq. reveals tissue-specific expression of flavonoid biosynthesis genes
Source: BMC Genom Data. 2021 Jun 5;22:19. doi: 10.1186/s12863-021-00973-4 (PMC8180045; doi:10.1186/s12863-021-00973-4)
Supplement: Supplementary file 2 — Additional file 2: Supplementary Table 1. Expression level of candidate A. oxyphylla unigenes coding for key enzymes involved in flavonoid biosynthesis pathways. [file 12863_2021_973_MOESM2_ESM.docx]

**Additional file 2 Supplementary Table 1**. Expression level of candidate *A. oxyphylla* unigenes coding for key enzymes involved in flavonoid biosynthesis pathways.

| Name | Gene ID | FPKM | | | | |
| --- | --- | --- | --- | --- | --- | --- |
|  |  |  |  |  |  |  |
|  |  | early-fruit | middle-fruit | late-fruit | root | leaf |
| FLS | unigene139585 | 458.62 | 179.04 | 82.35 | 0.09 | 59.11 |
| F3’5’H | unigene117017 | 27.31 | 12.91 | 14.99 | 49.87 | 0.12 |
| ANR | unigene115607 | 1020.66 | 2113.57 | 1768.39 | 498.78 | 337.65 |
| HCT | unigene109484 | 4.55 | 6.74 | 2.05 | 3.14 | 8.03 |
| HCT | unigene109483 | 68.88 | 53.75 | 71.79 | 210.44 | 70.62 |
| 4CL | unigene108821 | 43.81 | 37.85 | 42.6 | 128.7 | 6.12 |
| 4CL | unigene108819 | 98.89 | 150.82 | 118.44 | 39.41 | 5.2 |
| 4CL | unigene108818 | 2.7 | 2.03 | 1.94 | 68.18 | 7.12 |
| C4H | unigene106999 | 51.68 | 34.58 | 131.28 | 60.8 | 0.37 |
| 4CL | unigene095558 | 10.83 | 25.16 | 19.73 | 7.38 | 23.21 |
| DFR | unigene092310 | 307.69 | 440.88 | 231.95 | 73.62 | 2.67 |
| PAL | unigene077933 | 12.25 | 22.33 | 18.37 | 12.17 | 1.38 |
| F3H | unigene076049 | 4797.64 | 6785.61 | 4773.57 | 2370.02 | 427.85 |
| ANS | unigene074219 | 911.45 | 1216.78 | 774.03 | 348.66 | 9.27 |
| HCT | unigene067708 | 53.92 | 45.31 | 53.43 | 26.37 | 75.53 |
| CHS | unigene065545 | 43.39 | 13.51 | 8.57 | 46.91 | 3.58 |
| CHS | unigene065542 | 1158.04 | 870.33 | 562.51 | 81.74 | 160.18 |
| CCoAMT | unigene053332 | 355.08 | 147.74 | 245.95 | 591.43 | 53.1 |
| 4CL | unigene050620 | 1.34 | 1.89 | 5.85 | 0 | 0 |
| CHI | unigene042446 | 1129.52 | 588.88 | 300.05 | 235.97 | 17.68 |
| 4CL | unigene023081 | 33.21 | 34.13 | 35.64 | 96.7 | 14.42 |
| 4CL | unigene023078 | 28.25 | 35.02 | 21.21 | 35.23 | 8.07 |
| FLS | unigene018259 | 15.21 | 0.97 | 0.1 | 23.87 | 0.19 |
| FLS | unigene018258 | 25.59 | 1.04 | 0 | 38.39 | 0 |
| PAL | unigene017890 | 165.85 | 211.45 | 167.33 | 409.88 | 56.49 |
| F3’5’H | unigene007819 | 7.61 | 1.53 | 1.58 | 41.96 | 3.93 |
| F3’5’H | unigene007815 | 116.68 | 12.14 | 11.78 | 219.54 | 153.46 |
